# Establishing Cleft and Craniofacial Interdisciplinary Clinics in Africa: Opportunities and Challenges

**DOI:** 10.1016/j.identj.2026.109744

**Published:** 2026-07-16

**Authors:** Prasad Nalabothu, Emad Ghabrial, Veerasathpurush Allareddy

**Affiliations:** Department of Pediatrics, Oral Health and Orthodontics, University Center for Dental Medicine UZB, Basel, Switzerland; Department of Oral and Craniomaxillofacial Surgery, University Hospital Basel, Basel, Switzerland; School of Dentistry, Orthodontics Department, University of Pretoria, Pretoria, South Africa; Department of Orthodontics, College of Dentistry, University of Illinois Chicago, Chicago, Illinois, USA

Across the African continent, children affected by cleft lip and palate are confronted not only with the functional consequences of a condition that is surgically correctable but also with systemic barriers that impede access to comprehensive, team-based care. While there has been an increase in access to primary cleft repair in several African countries as a result of global surgical initiatives, coordinated, multidisciplinary cleft systems remain underdeveloped.^1^ This represents both a critical opportunity and a complex challenge for health systems strengthening. As African countries advance towards universal health coverage (UHC), investment in cleft care should serve as a tracer condition for assessing whether health systems can deliver coordinated, specialist, and longitudinal services.^2^

## The burden and the opportunity

Congenital anomalies represent substantial amounts of childhood morbidity within sub-Saharan Africa, although there is limited knowledge regarding the prevalence of such congenital anomalies in the region.^3^ Cleft lip and palate is the most common congenital craniofacial difference, with a low early mortality rate but requiring staged, multidisciplinary management spanning speech therapy, orthodontics, audiology, and psychological support.^1^ Estimates are that 5 billion people globally do not have adequate access to safe and affordable surgical care.^4^ However, with cleft lip/palate surgical patients, surgical access alone is not the only factor in obtaining optimal outcome results. The attainment of optimal outcomes is contingent upon the delivery of coordinated, team-based care over an extended period, a challenge that Africa’s predominantly fragmented health systems are ill-equipped to meet.^2^

Several African nations have demonstrated the feasibility of this approach. Ethiopia, Kenya, and Nigeria have established cleft clinics in collaboration with academic institutions or nongovernmental organisations, attaining primary repair rates that are commensurate with regional targets.^5^, ^6^, ^7^ It has been demonstrated that with dedicated coordination, even settings with limited resources can achieve positive surgical outcomes. Integration of cleft care into existing platforms, such as the Expanded Programme on Immunization or maternal and child health weeks, can serve as a cost-effective strategy for the early identification and referral of cases.^8^ Early detection facilitates the provision of timely nutritional support, feeding counselling, and surgical planning, thereby reducing preoperative morbidity.^9^

## Persistent challenges

Despite advancements, significant gaps remain in the current model. The most pressing issue is workforce scarcity. Speech-language pathologists, orthodontists, and paediatric audiologists are in short supply across sub-Saharan Africa, and where they exist, they are concentrated in urban academic centres.^10^ In the absence of such professionals, children who have undergone timely primary repair may be susceptible to velopharyngeal insufficiency, persistent speech intelligibility deficits, and untreated malocclusions.^1^ These factors can compromise long-term functional outcomes and social participation.

A qualitative study from Nigeria highlighted that families often travel hundreds of kilometres for surgery, only to find no postoperative speech or orthodontic follow-up available locally.^6^ Financial protection remains inadequate. Although surgical repair may be donated or subsidised, indirect costs, including transport, accommodation, and lost wages, frequently limit completion of care pathways.^6^ In countries with national insurance schemes, cleft-related services such as speech therapy and orthodontics are inconsistently covered.^2^ Outcome monitoring beyond surgical volume remains limited. Few countries maintain cleft registries capturing speech outcomes, orthodontic access, or continuity of care. Without such data, policymakers cannot evaluate equity of quality beyond operative counts.^3^

## A path forward

Addressing these gaps necessitates deliberate system reform rather than isolated programme expansion. First, regional cleft networks, centred on a tertiary referral hospital with outreach to provincial facilities, have the capacity to extend specialist oversight without requiring full multidisciplinary teams at every site.^2^ Telemedicine support for speech assessment and orthodontic planning has demonstrated potential in African contexts.^10^ Second, task-sharing strategies can mitigate workforce shortages. The distribution of tasks to nonspecialist providers, such as Expanded Programme on Immunization nurses trained to screen for clefts and refer early or community health workers supporting feeding and adherence, has the potential to address workforce shortages while developing local capacity.^8^ Third, UHC benefit packages must explicitly include multidisciplinary cleft services beyond surgical repair.^4^ The imminent African Union initiative on surgical strengthening offers a valuable opportunity to integrate such services into national surgical, obstetric, and anaesthesia plans.^9^ Cleft care should be positioned as a measurable indicator of system maturity, tracking not only surgical throughput but also functional outcomes and financial protection.

## Conclusions

Cleft clinics in Africa are beyond surgical scale-up, and evaluating health systems for coordinated care reflects UHC principles. Investing in multidisciplinary cleft services addresses the needs of children born with this condition and creates integrated care models for children with chronic conditions. Cleft care should no longer be viewed as charitable surgery but as a benchmark of integrated and equitable health system performance.

## Conflict of interest

None disclosed.
